# Roles of metabolic regulation in developing *Quercus variabilis* acorns at contrasting geologically-derived phosphorus sites in subtropical China

**DOI:** 10.1186/s12870-020-02605-y

**Published:** 2020-08-25

**Authors:** Jun Yuan, Ningxiao Sun, Hongmei Du, Shan Yin, Hongzhang Kang, Muhammad Umair, Chunjiang Liu

**Affiliations:** 1 School of Agriculture and Biology, Shanghai Jiao Tong University, Dongchuan Rd. 800, Shanghai, 200240 China; 2 School of Design, Shanghai Jiao Tong University, 800 Dongchuan RD, Shanghai, China; 3 Shanghai Urban Forest Ecosystem Research Station, National Forestry and Grassland Administration, 800 Dongchuan RD, Shanghai, China; 4 Shanghai Yangtze River Delta Eco-environmental Change and Management Observation and Research Station, Ministry of Education, P. R. China, 800 Dongchuan RD, Shanghai, China; 5 Key Laboratory of Urban Agriculture, Ministry of Agriculture, 800 Dongchuan RD., Shanghai, China

**Keywords:** *Quercus variabilis*, Phospharite areas, Metabolome, Acorn development, Subtropical soils

## Abstract

**Background:**

Phosphorus (P) -rich soils develop in phosphorite residing areas while P-deficient soils are ubiquitous in subtropical regions. Little has been reported that how metabolites participate in the seed development and the processes involved in their coping with contrasting-nutrient environments.

**Results:**

Here we quantified the metabolites of *Quercus variabilis* acorns in the early (July), middle (August), late (September) development stages, and determined element (C, H, O, N, P, K, Ca, Mg, S, Fe, Al, Mn, Na, Zn, and Cu) concentrations of acorns in the late stage, at geologically-derived contrasting-P sites in subtropical China. The primary metabolic pathways included sugar metabolism, the TCA cycle, and amino acid metabolism. Most metabolites (especially C- and N-containing metabolites) increased and then decreased from July to September. Acorns between the two sites were significantly discriminated at the three stages, respectively, by metabolites (predominantly sugars and organic acids). Concentrations of P, orthophosphoric acid and most sugars were higher; erythrose was lower in late-stage acorns at P-rich sites than those at P-deficient sites. No significant differences existed in the size and dry mass of individual acorns between oak populations at the two sites.

**Conclusions:**

Oak acorns at the two sites formed distinct metabolic phenotypes related to their distinct geologically-derived soil conditions, and the late-stage acorns tended to increase P-use-efficiency in the material synthesis process at P-deficient sites, relative to those at P-rich sites.

## Background

Subtropical soils are often characterized by phosphorus (P), calcium (Ca), and magnesium (Mg) deficiencies, with iron (Fe) and aluminum (Al) enriched [1, 2]. However, there are often some P-rich ores mixed in the P-deficient sites in some subtropical areas of China, which leads to significant changes in P and other elements across the region [3, 4]. These contrasting-P sites generally give rise to plants with different stoichiometry characteristics [3, 5–8], which can affect the metabolism and formation of compounds within cells and organisms [9, 10]. The metabolome represents all of the small molecules in organisms at a given moment [11, 12]. In ecological and physiological studies, metabolomics is primarily employed to explore the physiological status of organisms in response to variable environments [13–15]. To date, non-target metabolomics has been applied in many field experiments to elucidate the effects of environmental changes on the composition of metabolites [16–18].

Since the concept of ionome is first presented in 2003 [19], it has been used in the analysis of the responses to environmental factors in different plants [20], such as Arabidopsis (*Arabidopsis thaliana*) [21], tea (*Camellia sinensis*) [22], rice (*Oryza sativa*) [23]. With response to the variation of the genetic, developmental and environmental factors, elements are always bound to biological molecules [24–26], which are involved in lots of metabolisms, including carbon and nitrogen metabolism [27, 28]. Hence, the integration of metabolomics with element variations has enhanced the understanding of phenotypic responses involving both physiological and molecular mechanisms to different spatiotemporal scales [16, 29].

Serving as a sink tissue, seeds can mobilize stored nutrients to support the nascent processes of seed germination and seedling establishment in the plant life cycle [30–32]. Based on metabolomics, under a certain environment condition, researches have been conducted on the seed development of different plants, such as maize (*Zea mays*) [33, 34], barley (*Hordeum vulgare*) [35], soybean (*Glycine max*) [35–37] and lotus (*Nelumbo nucifera*) [38]. However, the adaption of seed metabolomes during development to variable soil nutrients in the field experiments has been rarely studied, which would deepen our knowledge of the adaption mechanism of the in situ plant to the environment.

*Quercus variabilis* is an important deciduous broadleaved oak for ecology, economy, and culture in Eastern Asia. In previous studies, there were distinct stoichiometry characteristics in both the leaves and seeds of *Q. variabilis* populations growing at P-rich and P-deficient sites [3, 39]. Moreover, strong correlations between metabolites and stoichiometry characteristics of leaves were exhibited in *Q. variabilis* populations at the two sites [16]. Therefore, for this study, using developing *Q. variabilis* seeds as samples, the questions we aimed to address were: 1) How do the metabolite profiles of developing plant seeds differ between P-rich and P-deficient sites? 2) Which specific metabolites play key roles in the metabolic regulation of seeds, in coping with variable nutrients at P-rich and P-deficient sites? This study aims to fill the gaps in our understanding as relates to how in situ plants metabolically adapt to sites with contrasting nutrient availability.

## Results

### Morphological characteristics of developing acorns at P-rich and P-deficient sites

**Fig. 1:**
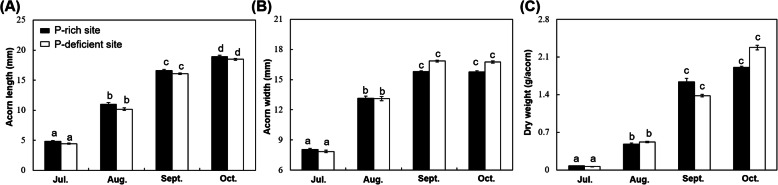
Variation of developing acorns at two sites. **a**, Length of acorns in July, August, September, and October growing at the two contrasting-P sites. **b**, Width of acorns in July, August, September, and October growing at the two contrasting-P sites. **c**, Dry weight of single acorn in July, August, September, and October growing at the two contrasting-P sites. Vertical bars indicate standard error of each mean (in July, *n* = 6 at P-rich and P-deficient sites; in August, September and October, *n* = 8 at P-rich and P-deficient sites). Different lowercase letters above the columns at P-rich site or P-deficient site means that length, width and dry weight were significantly different in acorns at different developmental stages under a particular soil condition (P-rich and P-deficient sites), respectively. There were no significant differences (*p* = 0.05) in acorns at P-rich and P-deficient sites at a particular developmental stage

The morphological characteristics of developing acorns at two contrasting-P sites were shown in Fig. 1: Acorn length significantly increased from July to October (*p* < 0.05) at both the P-rich and P-deficient sites (Fig. 1a); the width and dry mass of individual acorns significantly increased during developing, until reached the maximum values in September (Fig. 1b, c). Further, there were no significant differences in the acorn length and width, or the dry mass of single acorns between the P-rich and P-deficient sites at the four developmental stages (*p* > 0.05) (Fig. 1).

### Metabolomic changes in developing acorns

**Fig. 2:**
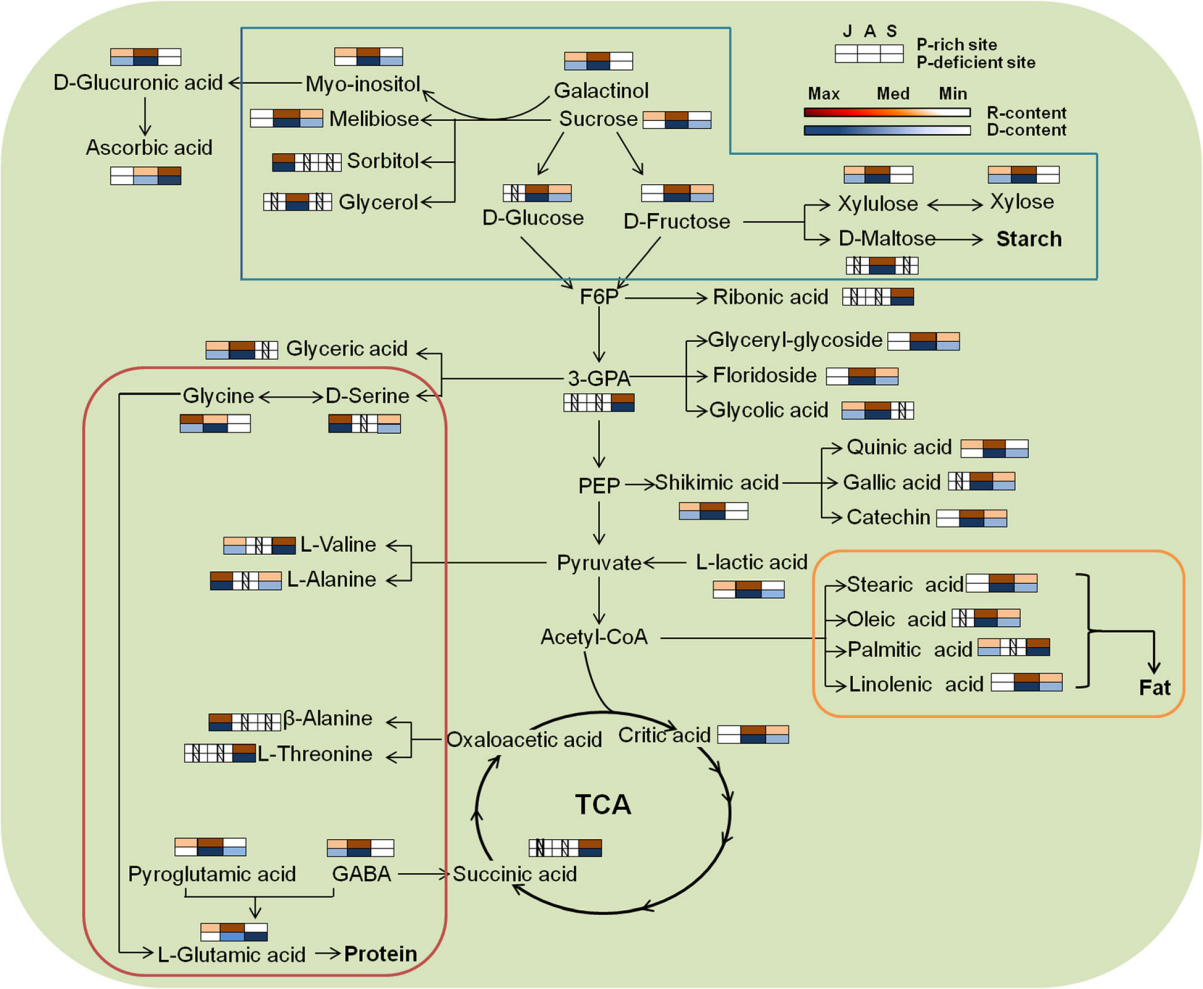
Metabolic map of acorn based on GC-MS. Pathways in the blue lined frame were included in starch synthesis; Pathways in orange lined frame were include in fat synthesis; Pathways in red lined frame were include in protein synthesis. GABA stands for γ-Aminobutyric acid, 3-GPA for glycerol-3-phosphate, N stands for not determined; R-content and D-content stand for metabolite content of P rich sites and P-deficient sites, respectively

A total of 100 metabolites were annotated and quantified at the three development stages, as shown in Table S1 (Additional file 4), which could be categorized into six groups based on the molecular structure: amino acids, sugars, organic acids, alcohols, amines, and esters. Additionally, a metabolic map of the process was developed based on the results of pathway analysis, which encompassed all of the metabolites of the three developmental stages (Fig. 2). The identified metabolites of the different developmental stages were different (Fig. 2). The central metabolic pathways of the acorns, including sugar metabolism, the TCA cycle, and amino acid metabolism, were observed in July, August, and September (Fig. 2; Additional file 5: Table S2). The target pathways were those with a pathway impact (PI) of > 0.1 (Additional file 5: Table S2). As shown in Table S2 (Additional file 5), β-alanine metabolism, pantothenate, and CoA biosynthesis were found in the acorns in July only.

**Fig. 3:**
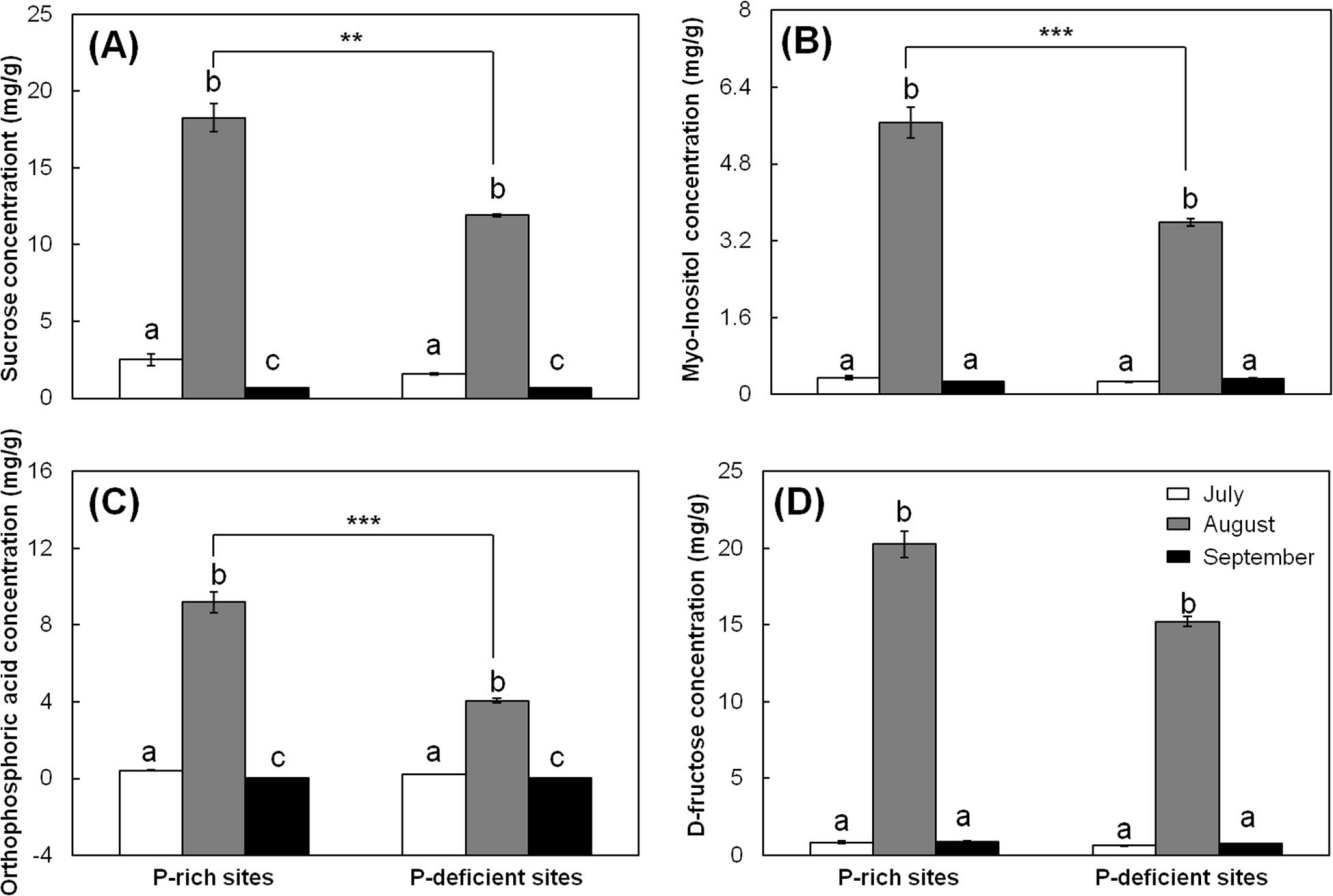
Concentrations of sucrose (**a**), Myo-inositol (**b**), orthophosphoric acid (**c**), and D-fructose (**d)** in acorns in July, August, and September of the two contrasting-P sites. Different lowercase letters above the columns at P-rich site or P-deficient site means that the metabolites were significantly different during different developmental stages under a particular soil condition (P-rich and P-deficient sites). ** means metabolites were significantly different (*p* < 0.01) in acorns at P-rich and P-deficient sites and *** means metabolites were significantly different (*p* < 0.001) in acorns at P-rich and P-deficient sites at a particular developmental stage

To better compare the metabolites of the acorns, we compared the concentrations of 47 metabolites (predominately sugars and organic acids), which were identified in acorns of all the three developmental stages (Additional file 2: Figure S2). Almost all of these 47 metabolites from the two sites of contrasting P availability were highest in August, except for glycine, d-mannopyranose, allose, glutaric acid, and ethanolamine (Additional file 2: Figure S2). The orthophosphoric acid, d-fructose, sucrose, and myo-inositol concentrations revealed obvious changes within the developing acorn at P-rich and P-deficient sites (Fig. 3; Additional file 4: Table S1): the concentrations of orthophosphoric acid and sucrose in acorns differed significantly among the three developmental stages at both the P-rich and P-deficient sites (*p* < 0.05); the concentrations of d-fructose and myo-inositol differed between the acorns in July and August, as well as acorns in August and September at the two sites (*p* < 0.05). Concretely, the concentrations of orthophosphoric acid, myo-inositol and d-fructose in the acorns in August were above 20 fold higher than those both in July and September at two sites; sucrose concentration in August were more than 7 fold higher than in July, and more than 25 fold higher than in September at the two sites (Fig. 3).

### Differential metabolites and discriminating elements in acorns between P-deficient and P-rich sites

Variance analysis of the metabolite concentration in developing *Q. variabilis* acorns at two contrasting-P sites revealed that the predominant metabolites of significant difference in the acorns of the two sites were glyceric acid, l-lactic acid, d-lyxose, and glycine in July, sugars (e.g., erythrose, allose, sucrose) and organic acids (e.g., glycine, linoleic acid, tartaric acid) in August, and sugars (e.g., erythrose, cellobiose, floridoside) in September (Fig. 5).

**Fig. 4:**
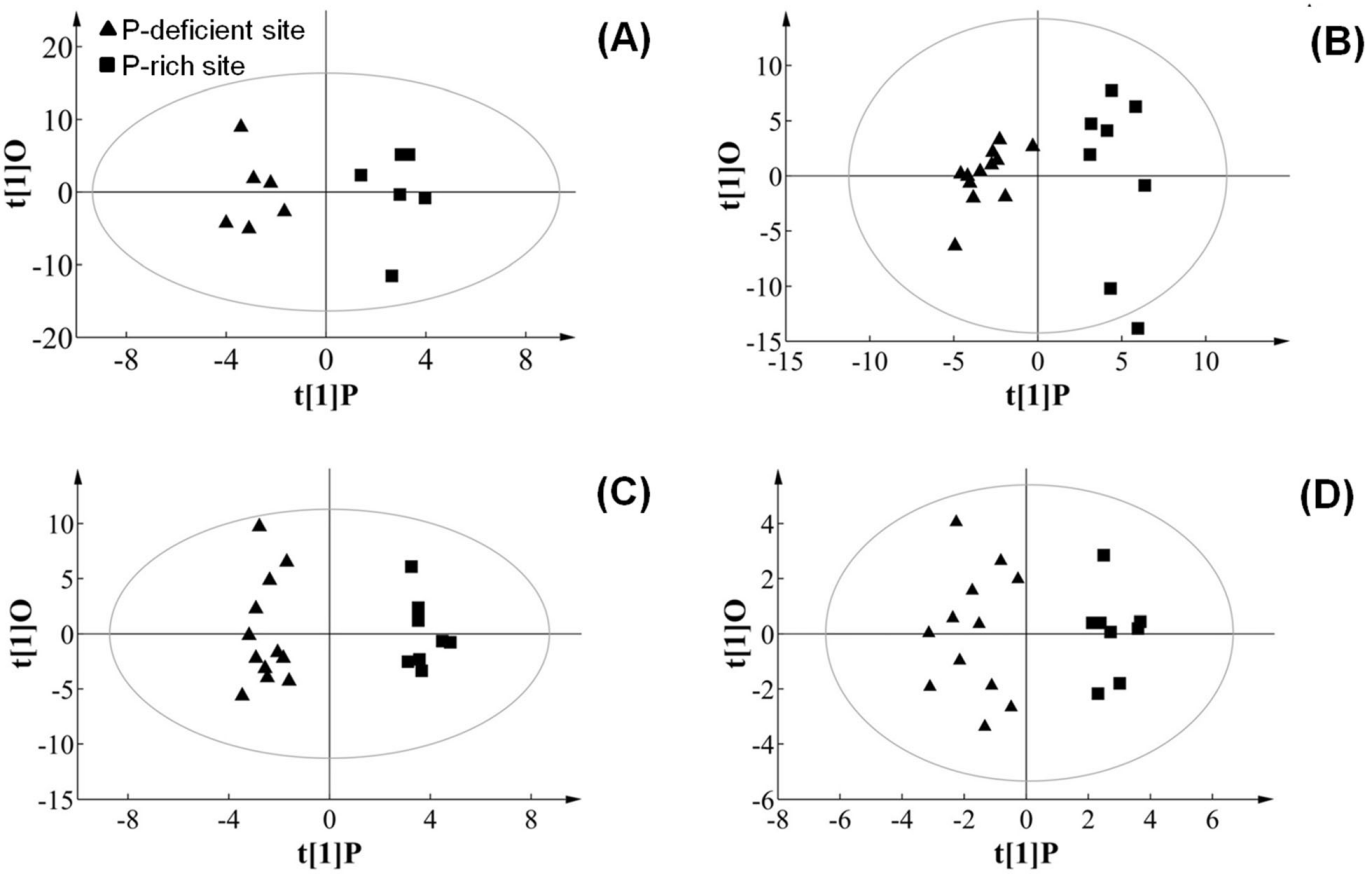
Score plots of orthogonal projections to latent structures discriminant analysis (OPLS-DA) of metabolomic and ionomic data. Metabolite data obtained from GC-MS of the acorns in July (**a**, R2Y [1] = 0.93, Q2 [1] = 0.45, CV-ANOVA *p* = 0.049), August (**b**, R2Y [1] = 0.74, Q2 [1] = 0.62, CV-ANOVA *p* = 0.018), and September (**c**, R2Y [1] = 0.85, Q2 [1] = 0.50, CV-ANOVA *p* = 0.005), and ionomic data obtained from element analysis and ICP-OES of acorns in September (**d**, R2Y [1] = 0.35, Q2 [1] = 0.64, CV-ANOVA *p* = 0.000) growing on two experimental sites with P-rich (squares) and P-deficient (triangles) content in soil

**Fig. 5:**
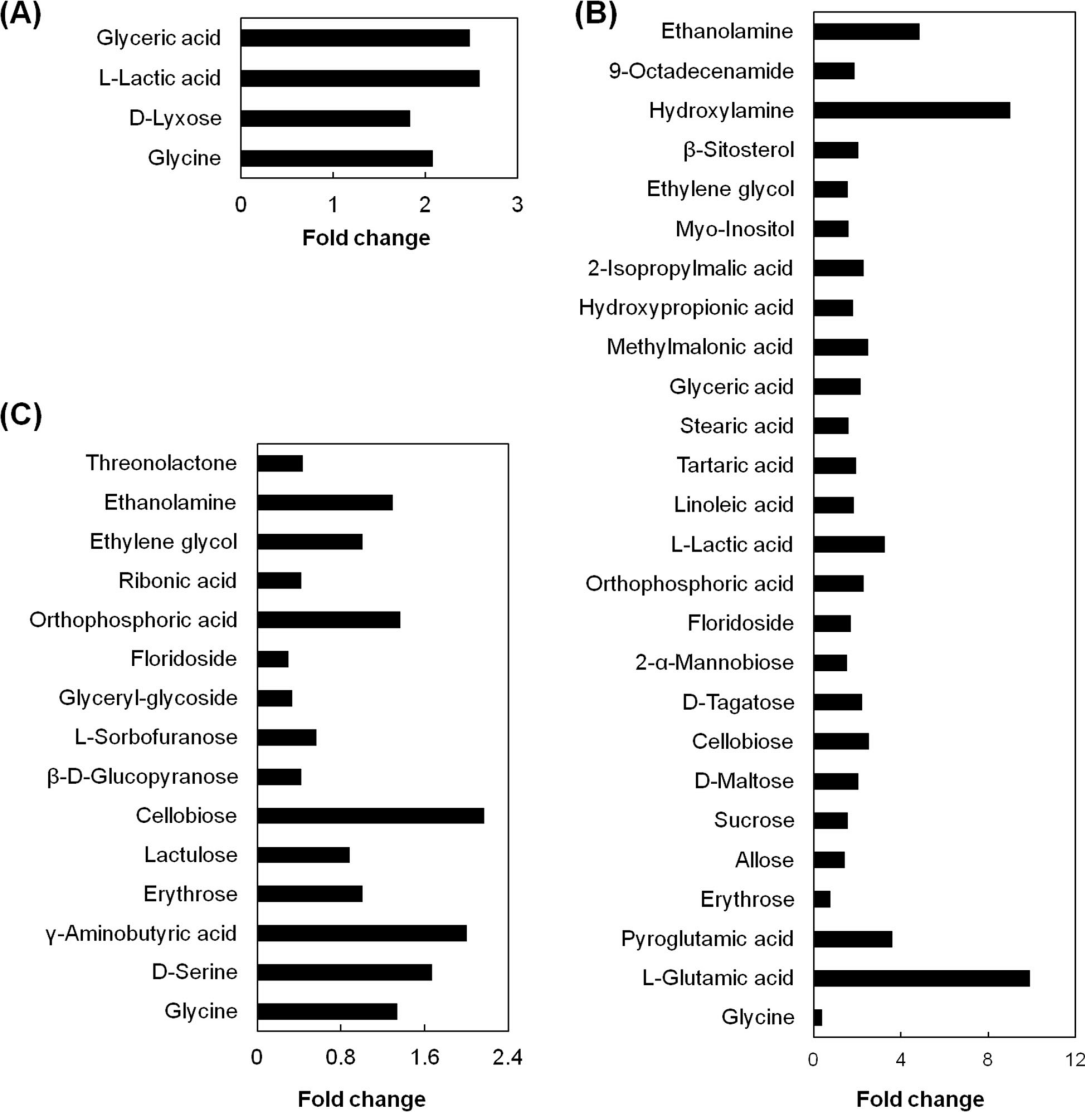
Variation of differential metabolites between the two contrasting-P sites in July (**a**), August (**b**), and September (**c**). Fold change stands for the proportion of metabolite concentrations in *Q. variabilis* seeds at P-rich sites to those at P-deficient sites the proportion of metabolite concentrations in *Q. variabilis* seeds at P-rich sites to those at P-deficient sites

According to the significant OPLS-DA model with R^2^ value of > 0.7 and Q^2^ value of > 0.5 (Additional file 3: Fig. S3A), the acorns of the two contrasting-P sites could be distinguished by metabolites (Fig. 4a) in July, with major contributions from four metabolites (glyceric acid, l-lactic acid, d-lyxose, and glycine). The concentrations of these metabolites were higher at the P-rich sites in contrast to the P-deficient sites (Fig. 5a; Additional file 4: Table S1). Similarly, based on significant OPLS-DA model with R^2^ value of > 0.7 and Q^2^ value of > 0.5 (Additional file 3: Figures. S3B, S3C), the acorns at the two contrasting-P sites were significantly discriminated by metabolites, with the major contributions from 26 metabolites in August (Fig. 4b, Fig. 5b), and 16 metabolites in September (Fig. 4c, Fig. 5c). In August, among the 26 metabolites, except for glycine and erythrose, other metabolites exhibited significantly higher concentrations at the P-rich sites in contrast to the P-deficient sites (*p* < 0.05), including sucrose and orthophosphoric acid (Fig. 5b; Additional file 4: Table S1). In particular, erythose concentration was higher at P-deficient sites, relative to the P-rich sites (Fig. 5b; Additional file 4: Table S1). In September, the 16 differential metabolites at the P-deficient sites were more abundant in contrast to those at the P-rich sites, including orthophosphoric acid. In particular, the acorn sugar (floridoside, glyceryl-glycoside, and β-d-glucopyranose) and organic acid (ribonic acid) concentrations at the P-deficient sites were two-fold higher compared with the P-rich sites (Fig. 5c; Additional file 4: Table S1).

**Fig. 6:**
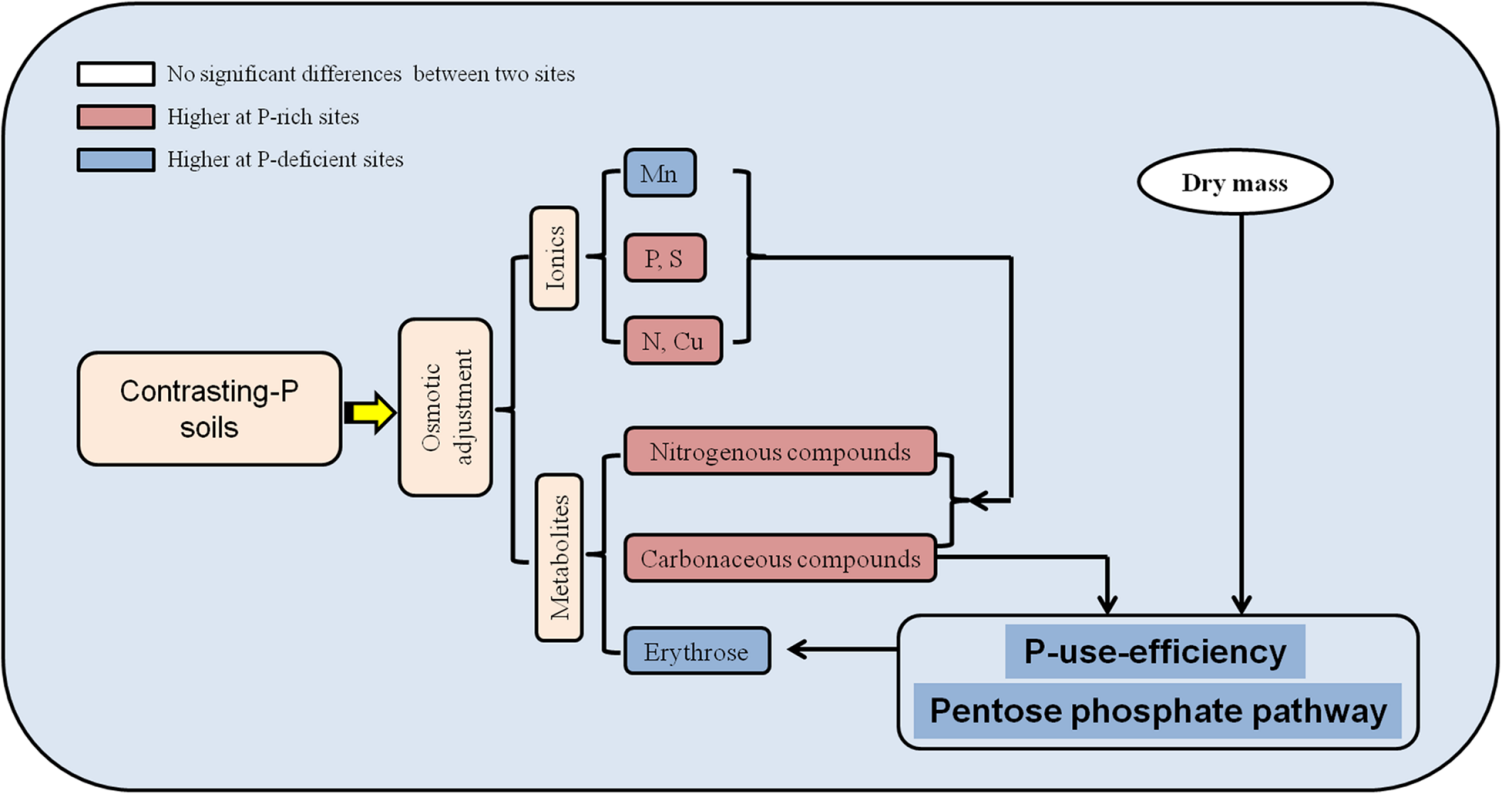
A proposed model for soil nutrition-induced primary metabolic changes in acorns of P-rich and P-deficient sites in September

Considering the physiological maturity of *Q. variabilis* acorns at the two sites was in September, when the dry mass of acorns reached stability during developing (Fig. 1), we also focused on metabolites-elements relationships of acorns in September. Based on OPLS-DA (Additional file 3: Fig. S3), the acorns from the P-rich and P-deficient sites were significantly discriminated via the ionomic data in September (Fig. 4c), with the major contributions from five elements (N, P, S, Mn, and Cu) (Fig. 6). Besides, concentrations of acorn N, P, S and Cu was higher, and acorn Mn was lower at P-rich sites than those at P-deficient sites (Fig. 6).

The relationships among these discriminating elements were complex (Additional file 7: Table S4): there were significantly positive correlations between N and P, between N and S, between P and S, and between Cu and S, and significantly negative correlations between N and Mn, and between Cu and Mn. Moreover, in September, acorn N and Mn showed complex correlations with acorn differential metabolites (Additional file 8: Table S5): acorn N was positively correlated with glycine (amino acid) and orthophosphoric acid, but negatively related to some sugars (e.g., l-sorbofuranose, β-d-glucopyranose, floridoside and glyceryl-glycoside); Mn was positively correlated with some sugars (e.g., β-d-glucopyranose, floridoside and glyceryl-glycoside), but negatively correlated with orthophosphoric acid (*p* < 0.05) (Fig. 6). Besides, P and S were positively related to glycine, negatively related to some sugars (e.g., floridoside and glyceryl-glycoside), and Cu was negatively related to some sugars (e.g., Lactulose, β-d-glucopyranose, floridoside and glyceryl-glycoside) in September (Additional file 8: Table S5; Fig. 6).

## Discussion

To the best of our knowledge, this study was the first to demonstrate how in situ plants generate seeds by adapting to variable nutrient environments, in terms of the metabolome of developing seeds in subtropical regions, where P was limiting for plant growth and development. Based on our results, the discussions focused on differences in the metabolic profiles of acorns, and the identification of metabolites that played key roles in metabolic regulation at the contrasting P sites.

### Sequential dynamic in **C- and N-containing metabolites** during acorn development

Our results clearly revealed that, for the oak populations at the two sites of contrasting P availability, from July to September, concentrations of most sugars, amino acids, and organic acids in acorns initially increased and then decreased (Additional file 4: Table S1; Fig. 2, Additional file 2: Fig. S2), with PI values of the same metabolomic pathways in the three developmental stages different (Additional file 5: Table S2). These results suggested that, during developing, metabolic states of acorns changed based on the sequentially dynamic of C- and N- containing metabolites. This was consistent with the results of Wang et al. [38], who found that the metabolites and proteins involved in the development of Lotus (*Nelumbo nucifera*) seeds were sequentially dynamic. On one hand, this might be caused by the vital function of these metabolites in seed development. From July to August, acorns synthesized many C- and N- containing metabolites (e.g., amino acids, sugars, and organic acids) to synthesize the storage substances in September, when acorns reached the physiological maturation with the dry mass being relatively stable (Fig. 1) [40]. As reported by others, key metabolites (e.g., amino acids, sugars, and organic acids) were significantly altered in developing maize [41], lotus [38], and wheat [42], even in developing fruits, such as, medlar (*Mespilus germanica*) [26], navel oranges (*Citrus sinensis*) [43], and ponkan (*Citrus reticulata*) [44].

On the other hand, the sequentially dynamic of metabolites in developing acorns at the contrasting-P sites might be related to the ordered regulation of genes in metabolite synthesis during plant development. The significant reduction of C- and N- containing metabolites was regulated by gene expression [45]. Although the most active genes in seeds were shared throughout development, each developmental stage utilized a set of genes that was up-regulated compared with other stages, or special at the GeneChip level [46]. Similarly, the transcriptome research of developing acorns by Miguel et al. [47] revealed that, carbohydrate metabolism, including sugar metabolism, the TCA cycle, and amino acid metabolism, was most represented during acorn development, but every stage had special up-regulated metabolisms, such as carbohydrates and energy metabolism for the middle stage. Similarly, even though some special metabolisms appeared in July, sugar metabolism, the TCA cycle, and amino acid metabolism comprised the core metabolic pathways in developing acorns (Fig. 2; Additional file 5: Table S2), with the size and dry mass increasing significantly from July to September at both P-rich and P-deficient sites (*p* < 0.05) (Fig. 1).

Besides, as a storage organ, acorns primarily accumulated starch (46.3–68.6%), followed by fats and proteins [48]. Therefore, during acorn development at the two contrasting-P sites, noticeable changes occurred in the concentrations of sugars, sugar alcohols, and amino acids, and the changes were related to their functions in the synthesis of starch and proteins (Fig. 2). Firstly, as a form of energy for plants, starch steadily accumulated during acorn development [49], resulting in the remarkable increase of acorn dry mass (Fig. 1). During this process, small sugar molecules (particularly sucrose and d-fructose) were converted to large sugar molecules (e.g., raffinose and starch) [38, 41], coupled with the consumption of sugar alcohols (e.g., myo-inositol and galactitol) (Fig. 2) [50], or acted as vital regulators [51, 52]. The accumulated large sugar molecules would serve as energy reserves (Fig. 2) [53], which could provide energy materials in the establishment of seedlings. Hence, at the two sites, during maturation process (from August to September), concentrations of sucrose and d-fructose, as well as myo-inositol and galactitol decreased significantly (Figs. 3, Additional file 2: Figure S2; Additional file 4: Table S1). Secondly, amino acids, which were generally synthesized in leaves, could be transported into seeds for further protein synthesis [54], and acted as intermediates for glycolysis and the TCA cycle in energy metabolism (Fig. 2) [55–57]. Hence, the decrease of most amino acids (particularly l-glutamic acid) during maturation (Additional file 2: Figure S2; Additional file 4: Table S1) might have been caused by the incorporation of amino acids in the synthesis of storage proteins.

### Differential C- and N-containing metabolites in oak acorn populations at P-rich and P-deficient sites

Our results clearly showed that metabolites, predominantly sugars and organic acids, differed significantly in acorns of oak populations at the P-rich and P-deficient sites (Fig. 5). This suggested that these types of sugars and organic acids were involved in the responses of in situ plant seeds to the variable supplies of soil nutrients due to geologically-derived environments. This had been proved by Ji et al. [16], who found that sugars and organic acids of *Q. variabilis* leaves played predominant roles in clearly discriminating *Q. variabilis* trees at the contrasting geologic-P sites. The vital functions of sugars and organic acids were also showed in other studies on the response of plant leaves and seeds under different nutritional conditions. Yan et al. [23] revealed that the added P could increase concentrations of sugars in oilseed flax seeds. P deficiencies [22] and Zn stress [58] predominantly altered concentrations of sugars and organic acids in tea (*Camellia sinensis*) leaves.

As the reproductive organ of *Q. variabilis* trees, acorns were characterized by the enrichment of starch during the development process [49]. Carbohydrate metabolism, including glycolysis, gluconeogenesis, amino sugar and nucleotide sugar metabolism, served as the essential biochemical process (Fig. 2) [47]. As two vital forms of metabolites, sugars and organic acids hold a larger percentage in plants than other metabolites, and sugars (e.g., fructose, sucrose, glucose) and organic acids (e.g., shikimate, glycine, glutamate) were the primary intermediates that participated in the carbon and nitrogen metabolism of plant seeds (Fig. 2) [38]. Similarly, the important functions of sugars and organic acids were observed in developing fruits, such as ponkan (*Citrus reticulata*) [59], navel oranges (*Citrus sinensis*) [43], and medlar (*Mespilus germanica*) [60].

### Increased efficiency of P use in the late stage acorns at P-deficient sites over P-rich sites

In September, the dry mass of *Q. variabilis* acorns reached stable (Fig. 1). We found that there were distinct metabolic shifts in acorns of this stage at such contrasting geologic-P sites (Fig. 6). The distinct metabolic shift of plants under different nutrient supply situation has been reported in other studies. Christian and Oliver [61] showed that *Chlamydomonas reinhardtii* developed highly distinctive metabolite profiles under N, P, S, or Fe deficiency conditions. Gargallogarriga et al. [62] revealed that increased P availability could lead to shifts in the metabolome through higher investments in the protection mechanisms of plants.

Concretely, our results indicated that the utilization of P was different to guarantee the same dry mass in acorns at P-rich and P-deficient sites with different soil nutrient supply (Fig. 6). Erythrose could be synthesized by erythrose-4-phosphate, which was an important intermediate product of the pentose phosphate pathway [63, 64], and the pentose phosphate pathway consumed less inorganic P than glycolysis [65]. In our study, the concentrations of most sugars, most organic acids, orthophosphoric acid, and P were lower, and erythrose was higher in the late-stage acorns at P-deficient sites than those at P-rich sites. But the dry mass and C concentration showed no significant differences in late-stage acorns at the two sites (Fig. 6; Additional file 6: Table S3). Unlikely, seeds of some Proteaceae species were considered to accumulate comparatively higher P concentration in seeds to adapt to low P environments [66], and produce small seeds in such P-impoverished habitats [67]. P addition could enhance the dry mass of maize (*Zea mays*) [68]. Besides, shifts in P usage were associated with variable P supplies to plants [69–71]. Hence, late-stage acorns at P-deficient sites might boost the pentose phosphate pathway to decrease P quotas through bypassing P consumption in glycolysis reactions, which increased the efficiency of P use in acorns at P-deficient sites (Fig. 6).

Further, similar to previous reports concerning the element concentrations of other organs [25, 72], the protein/nucleic-related (e.g., N, P, and S) and enzyme-related (e.g., Mn, Cu) elements of late-stage acorns were more highly impacted, in contrast to other types of elements at P-rich and P-deficient sites in the present study (Fig. 6). However, unlike our results, supply of P could enhance the concentrations of macro-elements (e.g., N, P, and S) in crops, such as urdbean (*Vigna mungo*) [73], wheat (*Triticum aestivum*), maize (*Zea mays*), and faba bean (*Vicia faba*) [74], but decrease the concentrations of micro-elements (e.g., Mn and Cu) in rice (*Oryza sativa*) [75]. Considering the strongly close relationships between these elements (e.g., N, P, S, Mn, and Cu) and metabolites in plants (Fig. 6) [76], and the vital effects of these elements on the synthesis of storage substances in plant seeds and the yield of plant seeds (Fig. 6) [75, 77, 78], it also could prove the distinct metabolic shifts in the late-stage acorns at the two sites.

## Conclusions

Metabolic regulation is one of the essential mechanisms for plants to generate formal seeds under the in situ conditions, by adapting to stresses due to nutrient deficiencies in soils, particularly in geologically-derived nutrient limited areas. In the present study, our results characterized the roles of metabolic regulation in the development of woody plant seeds at contrasting P sites in subtropical regions where soils were featured with deficient P, Ca and Mg. First, sugar metabolism, the TCA cycle, and amino acid metabolism were vital for the development of oak acorns due to their involvement in material synthesis. Moreover, the metabolites involved were sequentially dynamic, from July to September, with the most significant changes observed in the concentrations of orthophosphoric acid, d-fructose, sucrose, and myo-inositol. This demonstrated the characteristics of energy and nutrient accumulation in oak seeds. Second, there were significant differences in the acorns of oaks between the P-rich and P-deficient sites in terms of metabolites (predominantly sugars and organic acids) and elements, while no differences were observed in the size and the dry weight of individual acorns between P-rich and P-deficient sites. This verified the inconsistent between morphologies and chemical compositions of acorns in oak populations of the two site types. Third, in the late-stage (September) acorns, the concentrations of P, orthophosphoric acid and most sugars (particularly D-fructose and sucrose) were significantly higher, and erythrose was lower, at the P-rich sites in contrast to those at the P-deficient sites. This suggested that the late-stage acorns tended to increase the efficiency of P use in the process of material synthesis at the P-deficient sites relative to these at the P-rich sites. Our findings deepen our understanding of how in situ plants adapt to variable nutrient environments through metabolic mechanisms in subtropical regions.

## Methods

### Study sites

This study was based on a field experiment. The P-rich sites develop on phosphate rocks with high P content, locating in Kunming City (latitude 24°58′54.38″N, longitude 102°26′47.58″E, altitude 1869 m a.s.l), whereas the P-deficient sites develop on non-phosphate rocks with low P content, locating at Mouding, in Chuxiong City (latitude 25°14′48.25″N, longitude 101°32′35.62″E, altitude 1846 m a.s.l), of Yunnan Province, China. The stands at the P-rich (Kunming) and P-deficient (Mouding) sites are natural forests, which have been left anthropogenically untouched through history [79]. So, the trees we selected were the same age. Further, the chemical compositions of these phosphate and non-phosphate rocks have been revealed by Ji [80]. The level of P_2_O_5_ in the phosphate rocks is significantly higher over the non-phosphate rocks. The total P concentration are 2.23 mg g^− 1^ at P-rich sites and 0.3 mg g^− 1^ at P-deficient sites, while the available P concentration are 0.045 mg g^− 1^ at P-rich sites and 0.007 mg/g at P-deficient sites [8].

The climates of the two sites are similar, with the average annual temperatures of 16.0 °C at the two sites and the mean annual precipitation of 909.40 mm at Mouding, and 978.00 mm at Kunming in 2017. Figure S1 (Additional file 1) depicts the average monthly temperatures and precipitation of Mouding and Kunming, from 1981 to 2010, as well as the precipitation of the two sites, which occurred primarily during July and August (Additional file 1: Figure S1).

The chemical compositions of the soil from P-rich sites at Kunming and P-deficient sites at Mouding have been revealed by Wen et al. [8] and Ji et al. [80]. The pH of the soils at the two sites was ~ 5.22, with the soil C, N, P, K, Mg, and Mn concentration being much higher at the P-rich sites, in contrast to the P-deficient sites [16, 80]. Contingent on the location and extent of the *Q. variabilis* stands, six P-rich sites (20 m × 20 m) and six P-deficient sites (20 m × 20 m) were established in July. In August and September, eight P-rich sites (20 m × 20 m) and 12 P-deficient sites (20 m × 20 m) were established, respectively, where each site contained three plots.

### Sampling

Wild acorns were sampled from *Quercus variabilis* populations at P-rich (Kunming) and P-deficient (Mouding) sites in central Yunnan, China, where *Q. variabilis* trees were typically distributed. No permission was required to collect these acorn samples, and the study had no impact on the biological diversity of central Yunnan. Prof. Chunjiang Liu undertook the formal identification of samples at the two sites. In the P-rich site stand, the height of the *Q. variabilis* trees ranged from 17.67 m to 31.67 m, and the DBH (diameter at breast height) spanned 21.74 cm to 48.41 cm. In the P-deficient site stand, the height of *Q. variabilis* trees ranged from 22.50 m to 25.00 m, with the DBH spanning 16.03 cm to 28.66 cm. Four stages of acorn development, early stage (July), middle stage (August), late stage (September) and the last stage (October), were established, based on variations of developmental characteristics and the content of major substance (starch). Acorns were completely covered by the cupule in July, which was visible and in green in August, but became brown in September, and fell in October. Besides, in July and August, the accumulation rate of starch was much faster than other developmental stages, and in September, the starch content reached the maximum [49, 81]. So, morphological characteristics of *Q. variabilis* acorns were analyzed at the four developmental stages, but the metabolite profiling in acorns of July, August, and September were analyzed. In 2017, six acorns were randomly collected from three trees at each oak plot in the middle of July, August, September, and October, respectively. Parts of cotyledon from nine non-parasitized and peeled acorn samples from a given site were formed a composite sample in July. Parts of cotyledon from three non-parasitized and peeled acorn samples from a given plot were formed a composite sample in August and September. Voucher specimens were deposited in a public herbarium in School of Agriculture and Biology of Shanghai Jiao Tong University. To conduct GC-MS analysis, the acorns in July, August and September were immediately frozen in dry ice and stored at − 80 °C. The length, width, and dry weight of individual acorns at the four developmental stages were determined.

### Extraction, derivation, and analysis of acorn metabolites

Metabolite profiling analysis was performed using gas chromatography-mass spectrometry (7890A-5975C, Agilent, USA). The metabolite extraction technique was modified from Wu et al. [25] and Du et al. [82]. After composite acorn samples were defrosted, a 100 mg quantity of the fresh composite sample was introduced into a 2.0 mL centrifuge tube. Subsequently, 0.8 mL methanol-chloroform (3:1, v/v) and 30.0 μL of ribitol (2.0 mg mL^− 1^ stock in water) was introduced into each tube as an internal quantitative standard. The mixture was ground at 60.0 HZ for 80 s and centrifuged at 1200 r min^− 1^. for 10 min. 400.0 μL of the polar phase sample (aqueous and organic) was collected independently into 1.5 mL HPLC glass vials and dried in a benchtop centrifugal concentrator (Labconco Corporation, Kansas City, MI) for 3.0 h. Once the upper polar phase was thoroughly dried, methoximation, incubating the dried fraction at 37 °C for 1.5 h with 80.0 μL 15 mg mL^− 1^ methoxyamine hydrochloride, were carried out at first, and then trimethylsilylation, incubating the dried fraction at 70 °C for 1.0 h with 80.0 μL TMCS (BSTFA: TMCS = 99:1), was conducted.

The GC-MS analysis was carried out according to Du et al. [82]. The derivatization of samples was carried using a PerkinElmer gas chromatograph and TurboMass-Autosystem XL mass spectrometer (PerkinElmer lnc., Waltham, MS). A 1 μL aliquot of each sample was injected into a DB-5MS capillary column (30 mm × 0.25 mm × 250 mm) (Agilent JW Scientific, Folsom, CA). Following a solvent delay of 5 min, the GC oven temperature was adjusted to 60 °C; after injection for 1 min, the temperature of oven was raised from 60 °C to 300 °C at 5 °C min^− 1^ for 27 min. The injector and ion source temperatures were adjusted to 280 °C and 230 °C, respectively. Helium was applied as the carrier gas at a constant rate of 1.0 mL min^− 1^. Measurements were achieved with an electron impact at 70 e and at full scan mode, with a mass scan range of 33–600 m z^− 1^.

The metabolite levels were determined by the mass spectral library (2011) in MSD ChemStation (version E.02.02.1431; Agilent Inc., CA, USA), the National Institute of Standards and Technology (NIST), and ChromaTOF (version 4.50.8.0; Leco Corporation, MI, USA). The raw files were converted to NetCDF format, and then sequentially processed by ChromaTOF (version 4.50.8.0; Leco Corporation, MI, USA) for correcting the baseline, searching peaks, deconvoluting spectrum, and aligning compounds of the different samples. A number of artificial peaks were removed from the dataset based on the mass spectra and retention index (RI) comparison to the NIST library. The metabolites were expressed in the peak area, then normalized to the area of the internal standard ribitol for further analysis.

### Elemental analysis

In September, the oven-dried composite sample from a given plot was ground for element analysis, where 5 mg samples were used to analyze the total C, N, and H concentrations using an elemental analysis-stable isotope-ratio mass spectrometer (Vario ELIII; Elementar, Germany); 3 mg samples were used for total O concentration analysis via an Elemental Analyzer (Vario EL Cube; Germany). Total concentrations of P, K, Ca, Mg, S, Fe, Al, Mn, Na, Zn, and Cu were analyzed via acid digestion under high temperature. Briefly, 100 mg samples were introduced into 50 mL beakers, then added 3 mL of nitric acid and 0.5 mL of perchloric acid in them. After left undisturbed overnight (~ 12 h), they were digested on an electric heating plate until the composite liquid becoming clear (~ 6 h). The digested portions were placed into 15-mL flasks, to which distilled water (10 mL) was added. Subsequently, the plasma optical emission spectrometer (ICP-OES) (Iris Advantage 1000; Thermo Jarrell Ash, Franklin, MA) was used to determine concentrations of these elements at the Instrumental Analysis Center, Shanghai Jiao Tong University.

### Statistical analysis

The pathway analysis was carried out with the online software MetaboAnalyst 4.0 (http://www.metaboanalyst.ca/faces/ModuleView.xhtml). According to the results of the pathway analysis, the potential metabolic target pathways, with the value of pathway impact value (PI) > 0.1, were filtered out from the pathway topology. Analysis of variance [83] was performed to evaluate the differences of metabolites levels between groups with SPSS 20.0 (SPSS Inc., USA). The SIMCA-P version 14.1 (Umetrics, Sweden) was utilized to assess the effects of different types of geological P on the metabolic data and ionomic data by conducting supervised orthogonal partial least squares discrimination analysis (OPLS-DA). Permutation tests (200) or ANOVA of the cross-validated residuals (CV-ANOVA) were conducted to validate the OPLS-DA model. A R^2^ value of > 0.7 and a Q^2^ value of > 0.5 in OPLS-DA denoted that the models were highly significant [84]. Furthermore, the variable importance in projection (VIP) was critical for explaining the data obtained with OPLS-DA. Metabolites with a VIP of above 1.0 and a *p* value of below 0.05, were selected as differential metabolites and discriminating elements (biomarkers), which played a larger role in distinguishing acorns growing at two sites having P-rich and P-deficient soils. Pearson’s correlation test was performed to analyze the relationships among discriminating elements, and relationships of discriminating elements and differential metabolites of acorns in September with SPSS 20.0 (SPSS Inc., USA). All data were log_10_-transformed to improve normality prior to analysis.

## Supplementary information

**Additional file 1: Figure S1.** Mean monthly temperature and mean monthly precipitation of Mouding and Kunming, from 1981 to 2010.

**Additional file 2: Figure S2.** Variation of the same metabolites in acorns during three developmental stages at P-rich and P-deficient sites. A, Overlap of all metabolites identified in acorns during three developmental stages. R&D, at P-rich and P-deficient sites. B, Heatmap analysis of the same metabolites identified in acorns during three developmental stages. Values were normalized with the Z-transformation. PR, P-rich sites; PD, P-deficient sites; J, July; A, August; S, September.

**Additional file 3: Figure S3.** Over-fitting of OPLS-DA model validation based on metabolomic and ionomic data of developing acorns.

**Additional file 4: Table S1.** All metabolites identified in acorns at P-rich and P-deficient sites in July, August and September (mg g^− 1^). --, not determined. All data are mean ± SE (in July, *n* = 4 of P-rich sites, *n* = 5 of P-deficient sites; in August and September, *n* = 8 of P-rich sites, of P-deficient sites, *n* = 12).

**Additional file 5: Table S2.** Results of pathway analysis involving all identified metabolites in acorns during three developmental stages. All pathways shown in the table are potential target metabolic pathways with pathway impacts of above 0.1.

**Additional file 6: Table S3.** Concentrations of acorn elements in September at P-rich and P-deficient sites (mg/g). All data are mean ± SE (*n* = 8 at P-rich sites; *n* = 12, at P-deficient sites). Discriminating elements, with bold text for *p* value and VIP (variable importance projection plot) value, were selected based on the *p* < 0.05 from the t test (Significance, *p* < 0.05) and VIP > 1 from OPLS-DA models.

**Additional file 7: Table S4.** Correlations (*r*, Pearson’s correlation coefficient) between acorn discriminating elements between P-rich and P-deficient sites in September. Significance, *p* value < 0.05: ^*^, *p* < 0.05; ^**^, *p* < 0.01.

**Additional file 8: Table S5.** Correlations (*r*, Pearson’s correlation coefficient) between differential metabolites and discriminating elements in acorns between P-rich and P-deficient sites in September. Significance, *p* value < 0.05: ^*^, *p* < 0.05; ^**^, *p* < 0.01.

## Data Availability

The original dataset of metabolites is available from the Metabolights database (https://www.ebi.ac.uk/metabolights) with study identifier MTBLS888.
