# Supplementary material for: Roles of metabolic regulation in developing Quercus variabilis acorns at contrasting geologically-derived phosphorus sites in subtropical China
Source: BMC Plant Biol. 2020 Aug 25;20:389. doi: 10.1186/s12870-020-02605-y (PMC7449008; doi:10.1186/s12870-020-02605-y)
Supplement: Supplementary file 1 — Additional file 1: Figure S1. Mean monthly temperature and mean monthly precipitation of Mouding and Kunming, from 1981 to 2010. [file 12870_2020_2605_MOESM1_ESM.doc]

**Figure S1** Mean monthly temperature and mean monthly precipitation of Mouding and Kunming, from 1981 to 2010
