# Supplementary material for: Roles of metabolic regulation in developing Quercus variabilis acorns at contrasting geologically-derived phosphorus sites in subtropical China
Source: BMC Plant Biol. 2020 Aug 25;20:389. doi: 10.1186/s12870-020-02605-y (PMC7449008; doi:10.1186/s12870-020-02605-y)
Supplement: Supplementary file 2 — Additional file 2: Figure S2. Variation of the same metabolites in acorns during three developmental stages at P-rich and P-deficient sites. A, Overlap of all metabolites identified in acorns during three developmental stages. R&D, at P-rich and P-deficient sites. B, Heatmap analysis of the same metabolites identified in acorns during three developmental stages. Values were normalized with the Z-transformation. PR, P-rich sites; PD, P-deficient sites; J, July; A, August; S, September. [file 12870_2020_2605_MOESM2_ESM.doc]

**Figure S2** Variation of the same metabolites in acorns during three developmental stages at P-rich and P-deficient sites. A, Overlap of all metabolites identified in acorns during three developmental stages. R&D, at P-rich and P-deficient sites. B, Heatmap analysis of the same metabolites identified in acorns during three developmental stages. Values were normalized with the Z-transformation. PR, P-rich sites; PD, P-deficient sites; J, July; A, August; S, September.
