# Supplementary material for: Roles of metabolic regulation in developing Quercus variabilis acorns at contrasting geologically-derived phosphorus sites in subtropical China
Source: BMC Plant Biol. 2020 Aug 25;20:389. doi: 10.1186/s12870-020-02605-y (PMC7449008; doi:10.1186/s12870-020-02605-y)
Supplement: Supplementary file 3 — Additional file 3: Figure S3. Over-fitting of OPLS-DA model validation based on metabolomic and ionomic data of developing acorns. [file 12870_2020_2605_MOESM3_ESM.doc]

**Figure S3** Over-fitting of OPLS-DA model validation based on metabolomic and ionomic data of developing acorns. Metabolite data obtained from GC-MS of the acorns in July (A, R2Y[1] = 0.93, Q2[1] = 0.45, CV-ANOVA *p* = 0.049), August (B, R2Y[1] = 0.74, Q2[1] = 0.62, CV-ANOVA *p* = 0.018) and September (C, R2Y[1] = 0.85, Q2[1] = 0.50, CV-ANOVA *p* = 0.005), and ionomic data obtained from element analysis and ICP-OES of acorns in September (E, R2Y[1] = 0.35, Q2[1] = 0.64, CV-ANOVA *p* = 0.000), growing on two experimental sites with P-rich (squares) and P-deficient (triangles) content in soil.

**
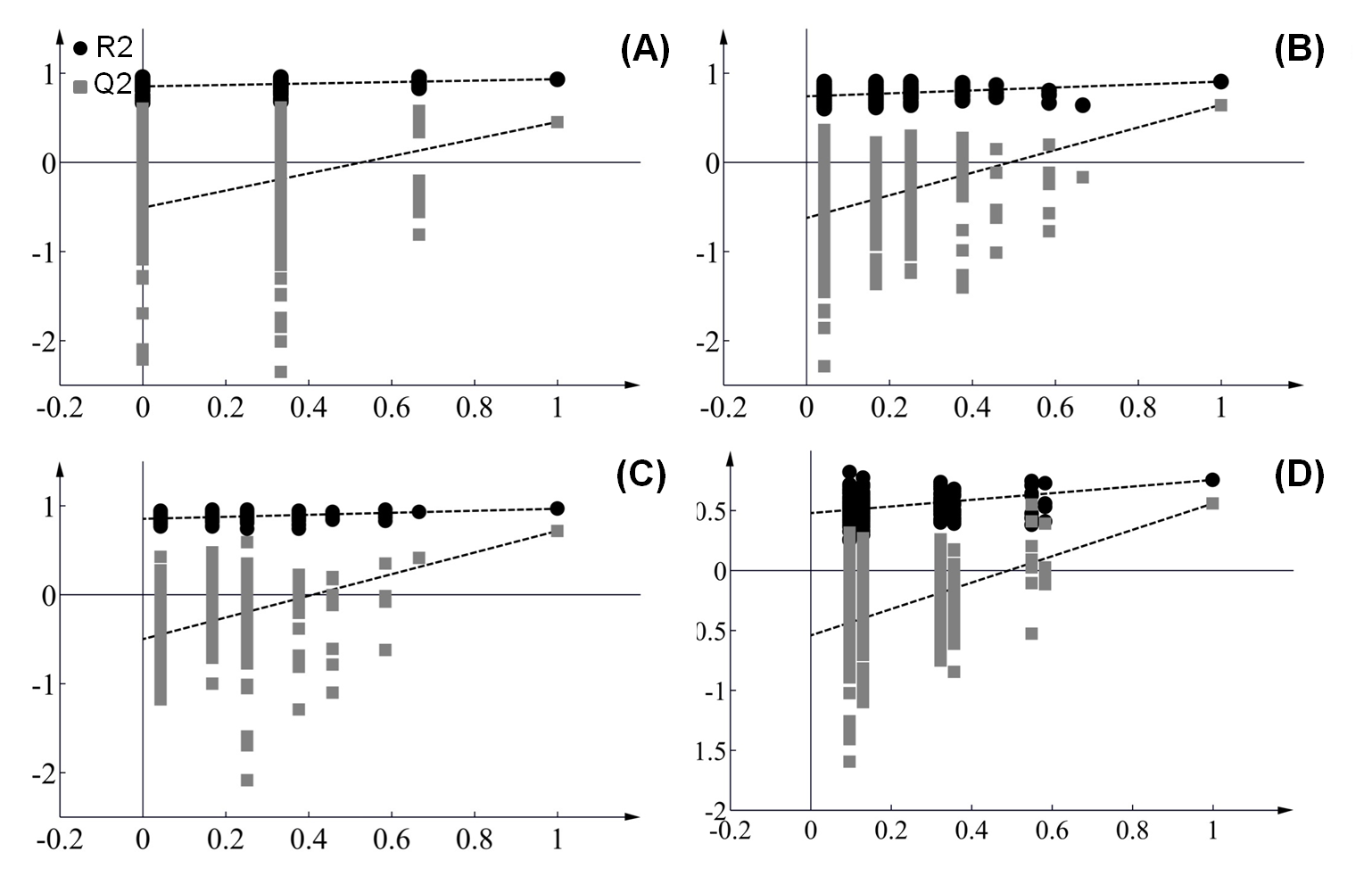
**
