# Supplementary material for: Roles of metabolic regulation in developing Quercus variabilis acorns at contrasting geologically-derived phosphorus sites in subtropical China
Source: BMC Plant Biol. 2020 Aug 25;20:389. doi: 10.1186/s12870-020-02605-y (PMC7449008; doi:10.1186/s12870-020-02605-y)
Supplement: Supplementary file 4 — Additional file 4: Table S1. All metabolites identified in acorns at P-rich and P-deficient sites in July, August and September (mg g− 1). --, not determined. All data are mean ± SE (in July, n = 4 of P-rich sites, n = 5 of P-deficient sites; in August and September, n = 8 of P-rich sites, of P-deficient sites, n = 12). [file 12870_2020_2605_MOESM4_ESM.doc]

**Table S1** All metabolites identified in acorns at P-rich and P-deficient sites in July, August and September (mg g-1)

| No. | Metabolite | Categorization | RT (min) | m/z |  | **P-rich site** |  |  |  | **P-deficient site** | |
| --- | --- | --- | --- | --- | --- | --- | --- | --- | --- | --- | --- |
|  |  |  |  |  | Jul. | Aug. | Sept. |  | Jul. | Aug. | Sept. |
| 1 | **Ethylene glycol** | Alcohols | 7.0244 | 147 | 0.0280±0.0045 | 0.3414±0.0162 | 0.0056±0.0000 |  | 0.0106±0.0003 | 0.2218±0.0023 | 0.0062±0.0000 |
| 2 | Dithiothreitol | Alcohols | 8.1315 | 117 | 0.0005±0.0001 | -- | -- |  | 0.0009±0.0000 | -- | -- |
| 3 | Propylene glycol | Alcohols | 8.3761 | 117 | 0.0031±0.0006 | -- | -- |  | 0.0036±0.0001 | -- | -- |
| 4 | **L-Lactic acid** | Organic acids | 8.8911 | 117 | 0.0723±0.0068 | 2.0155±0.1856 | 0.0448±0.0014 |  | 0.0279±0.0008 | 0.6220±0.0152 | 0.0341±0.0013 |
| 5 | Glycolic acid | Organic acids | 9.3416 | 147 | 0.0065±0.0009 | 0.0857±0.0045 | -- |  | 0.0024±0.0001 | 0.0962±0.0060 | -- |
| 6 | Acrylic acid | Organic acids | 9.6248 | 147 | 0.0009±0.0001 | 0.0234±0.0011 | -- |  | 0.0006±0.0000 | 0.0170±0.0005 | -- |
| 7 | L-Valine | Amino acids | 9.7343 | 55 | 0.0048±0.0010 | -- | 0.0072±0.0005 |  | 0.0026±0.0001 | -- | 0.0048±0.0002 |
| 8 | L-Alanine | Amino acids | 10.0368 | 73 | 0.0183±0.0040 | -- | 0.0054±0.0004 |  | 0.0151±0.0004 | -- | 0.0039±0.0002 |
| 9 | Hydroxypropionic acid | Organic acids | 11.0666 | 147 | 0.0031±0.0003 | 0.0726±0.0036 | -- |  | 0.0025±0.0001 | 0.0411±0.0008 | -- |
| 10 | Oxalic acid | Organic acids | 11.1954 | 147 | 0.2831±0.0525 | 3.3384±0.1964 | -- |  | 0.0877±0.0024 | 2.3500±0.0423 | -- |
| 11 | Malonic acid | Organic acids | 12.8303 | 147 | 0.0068±0.0008 | 0.0000±0.0000 | -- |  | 0.0058±0.0002 | -- | -- |
| 12 | Methylmalonic acid | Organic acids | 13.7701 | 73 | -- | 0.9207±0.0700 | -- |  | -- | 0.3715±0.0133 | -- |
| 13 | Diethylene glycol | Alcohols | 13.9245 | 117 | 0.0006±0.0001 | -- | -- |  | 0.0002±0.0000 | -- | -- |
| 14 | Urea | Amines | 14.0017 | 147 | 0.0086±0.0021 | -- | 0.0107±0.0005 |  | 0.0018±0.0001 | -- | 0.0070±0.0005 |
| 15 | **Ethanolamine** | Amines | 14.4716 | 73 | 0.0357±0.0057 | 0.0344±0.0038 | 0.0573±0.0010 |  | 0.0196±0.0005 | 0.0067±0.0010 | 0.0437±0.0008 |
| 16 | **Orthophosphoric acid** | Organic acids | 14.6390 | 299 | 0.4252±0.0419 | 9.1840±0.5481 | 0.0454±0.0008 |  | 0.2174±0.0060 | 4.0567±0.1273 | 0.0329±0.0004 |
| 17 | **1,2,3-trihydroxybutane** | Others | 15.1410 | 117 | 0.0017±0.0001 | 0.1449±0.0063 | 0.0019±0.0001 |  | 0.0024±0.0001 | 0.1084±0.0048 | 0.0044±0.0002 |
| 18 | **Glycerol** | Alcohols | 15.2311 | 117 | 0.0034±0.0003 | 0.1375±0.0083 | 0.0256±0.0007 |  | 0.0042±0.0001 | 0.0885±0.0042 | 0.0211±0.0004 |
| 19 | **Succinic acid** | Organic acids | 15.7654 | 147 | -- | 0.0000±0.0000 | 0.0868±0.0045 |  | -- | 0.0000±0.0000 | 0.0844±0.0037 |
| 20 | 2-Butenedioic acid | Organic acids | 15.8297 | 147 | 0.1947±0.0215 | 2.7776±0.1390 | -- |  | 0.1222±0.0034 | 1.8280±0.0721 | -- |
| 21 | Methylsuccinic acid | Organic acids | 16.1258 | 147 | 0.0013±0.0001 | -- | 0.0015±0.0001 |  | 0.0016±0.0000 | -- | 0.0015±0.0000 |
| 22 | Glyceric acid | Organic acids | 16.2095 | 147 | 0.0091±0.0010 | 0.0431±0.0035 | - |  | 0.0037±0.0001 | 0.0199±0.0004 | -- |
| 23 | Maleic acid | Organic acids | 16.7502 | 147 | -- | -- | 0.0057±0.0004 |  | -- | -- | 0.0091±0.0006 |
| 24 | D-Serine | Amino acids | 17.0076 | 73 | 0.0199±0.0031 | -- | 0.0147±0.0010 |  | 0.0148±0.0004 | -- | 0.0089±0.0003 |
| 25 | L-Threonine | Amino acids | 17.6577 | 117 | 0.0023±0.0004 | -- | 0.0057±0.0005 |  | 0.0011±0.0000 | -- | 0.0051±0.0003 |
| 26 | β-Alanine | Amino acids | 18.7262 | 147 | 0.0039±0.0008 | -- | -- |  | 0.0013±0.0000 | -- | -- |
| 27 | **Erythrose** | Sugars | 18.8100 | 103 | -- | 0.0300±0.0013 | 0.0015±0.0001 |  | -- | 0.0392±0.0005 | 0.0023±0.0001 |
| 28 | L-Threose | Sugars | 19.4085 | 117 | 0.0003±0.0000 | -- | -- |  | 0.0002±0.0000 | -- | -- |
| 29 | **Pyrimidine** | Others | 19.6274 | 103 | -- | 0.0364±0.0042 | 0.0004±0.0000 |  | -- | 0.0064±0.0004 | 0.0003±0.0000 |
| 30 | L-Pipecolic acid | Organic acids | 20.5350 | 75 | -- | -- | 0.0036±0.0002 |  | -- | -- | 0.0035±0.0001 |
| 31 | **Erythritol** | Alcohols | 20.5735 | 103 | 0.0009±0.0001 | 0.0206±0.0005 | 0.0021±0.0001 |  | 0.0008±0.0000 | 0.0191±0.0004 | 0.0030±0.0001 |
| 32 | D-Threitol | Alcohols | 20.7602 | 103 | 0.0021±0.0001 | 0.0656±0.0029 | -- |  | 0.0017±0.0000 | 0.0593±0.0018 | -- |
| 33 | **Pyroglutamic acid** | Organic acids | 21.0499 | 73 | 0.1639±0.0350 | 2.0489±0.1546 | 0.0853±0.0037 |  | 0.0487±0.0014 | 0.5727±0.0498 | 0.0651±0.0019 |
| 34 | **Hydroxylamine** | Amines | 21.2623 | 133 | -- | 0.0626±0.0091 | 0.0220±0.0005 |  | -- | 0.0070±0.0004 | 0.0201±0.0005 |
| 35 | 1,2,3-Butanetriol | Alcohols | 21.4297 | 73 | -- | 0.4168±0.0153 | -- |  | -- | 0.3454±0.0029 | -- |
| 36 | **γ-Aminobutyric acid** | Organic acids | 21.5777 | 147 | 0.0714±0.0150 | 0.5080±0.0758 | 0.0262±0.0017 |  | 0.0360±0.0010 | 0.1549±0.0079 | 0.0134±0.0004 |
| 37 | **Threonolactone** | Esters | 22.2857 | 147 | 0.0017±0.0002 | 0.3263±0.0319 | 0.0065±0.0002 |  | 0.0012±0.0000 | 0.2527±0.0054 | 0.0139±0.0007 |
| 38 | **α-Isopropylmalic acid** | Organic acids | 22.4144 | 147 | 0.0026±0.0002 | 0.0411±0.0029 | 0.0015±0.0000 |  | 0.0013±0.0000 | 0.0181±0.0004 | 0.0012±0.0000 |
| 39 | **Glycine** | Amino acids | 22.7000 | 133 | 0.0182±0.0015 | 0.0142±0.0014 | 0.0081±0.0002 |  | 0.0087±0.0002 | 0.0367±0.0008 | 0.0060±0.0001 |
| 40 | **Tartaric acid** | Organic acids | 22.8843 | 117 | -- | 0.1387±0.0105 | 0.0180±0.0010 |  | -- | 0.0716±0.0021 | 0.0162±0.0004 |
| 41 | **Glutaric acid** | Organic acids | 23.4250 | 73 | 0.0135±0.0029 | 0.0124±0.0010 | 0.0101±0.0004 |  | 0.0334±0.0009 | 0.0131±0.0006 | 0.0123±0.0003 |
| 42 | **L-Glutamic acid** | Organic acids | 23.5280 | 73 | 0.0283±0.0033 | 0.7323±0.0983 | 0.0852±0.0036 |  | 0.0151±0.0004 | 0.0737±0.0128 | 0.0683±0.0016 |
| 43 | D-Ribose | Sugars | 23.6953 | 129 | 0.0032±0.0004 | 0.1781±0.0082 | -- |  | 0.0021±0.0001 | 0.2122±0.0039 | -- |
| 44 | Ribonolactone | Others | 23.9850 | 73 | 0.0050±0.0004 | 0.0249±0.0016 | -- |  | 0.0063±0.0002 | 0.0206±0.0009 | -- |
| 45 | **D-Xylose** | Sugars | 24.2038 | 103 | 0.0087±0.0007 | 0.1507±0.0078 | 0.0049±0.0002 |  | 0.0048±0.0001 | 0.1234±0.0025 | 0.0044±0.0001 |
| 46 | **D-Lyxose** | Sugars | 24.3647 | 103 | 0.0334±0.0020 | 0.5457±0.0334 | 0.0170±0.0008 |  | 0.0182±0.0005 | 0.3840±0.0101 | 0.0176±0.0004 |
| 47 | D-Arabinose | Sugars | 24.6866 | 103 | 0.0538±0.0056 | 0.8281±0.1038 | -- |  | 0.0923±0.0026 | 0.7632±0.0612 | -- |
| 48 | **D-Xylulose** | Sugars | 25.2079 | 103 | 0.0085±0.0011 | 0.0953±0.0068 | 0.0041±0.0000 |  | 0.0064±0.0002 | 0.0625±0.0013 | 0.0042±0.0001 |
| 49 | **Levoglucosan** | Sugars | 25.5298 | 73 | 0.0194±0.0020 | 0.5663±0.0340 | 0.0018±0.0000 |  | 0.0146±0.0004 | 0.4401±0.0052 | 0.0023±0.0001 |
| 50 | **D-Rhamnose** | Sugars | 25.6263 | 117 | 0.0040±0.0004 | 0.0427±0.0016 | 0.0059±0.0002 |  | 0.0034±0.0001 | 0.0488±0.0045 | 0.0054±0.0002 |
| 51 | **L-Arabinitol** | Alcohols | 25.7550 | 103 | 0.0085±0.0007 | 0.2937±0.0192 | 0.0076±0.0003 |  | 0.0118±0.0003 | 0.3351±0.0089 | 0.0104±0.0004 |
| 52 | Glycerol-3-phosphate | Organic acids | 26.5404 | 103 | -- | -- | 0.0053±0.0002 |  | -- | -- | 0.0050±0.0001 |
| 53 | **D-Psicose** | Sugars | 26.6755 | 147 | 0.0453±0.0035 | 1.4265±0.1584 | 0.0045±0.0002 |  | 0.0472±0.0013 | 0.7981±0.0289 | 0.0045±0.0001 |
| 54 | **L-Sorbofuranose** | Sugars | 26.9329 | 73 | 0.0247±0.0032 | 4.0371±0.1852 | 0.1635±0.0060 |  | 0.0270±0.0007 | 5.1073±0.0817 | 0.2898±0.0125 |
| 55 | **D-Mannopyranose** | Sugars | 27.3063 | 45 | 0.0726±0.0090 | 1.3612±0.0505 | 4.0847±0.0879 |  | 0.0382±0.0011 | 1.1868±0.0186 | 3.9372±0.0359 |
| 56 | Ribonic acid | Organic acids | 27.4157 | 117 | -- | -- | 0.0052±0.0002 |  | -- | -- | 0.0120±0.0004 |
| 57 | **Shikimic acid** | Organic acids | 27.6281 | 73 | 0.0218±0.0016 | 0.4849±0.0223 | 0.0105±0.0010 |  | 0.0185±0.0005 | 0.6607±0.0176 | 0.0144±0.0013 |
| 58 | **Citric acid** | Organic acids | 27.7568 | 147 | 0.1390±0.0119 | 2.2145±0.2093 | 0.1806±0.0102 |  | 0.0792±0.0022 | 1.3478±0.0596 | 0.1908±0.0055 |
| 59 | D-Tagatose | Sugars | 28.2138 | 103 | 0.0315±0.0048 | 0.4899±0.0511 | -- |  | 0.0211±0.0006 | 0.2207±0.0071 | -- |
| 60 | **Quinic acid** | Organic acids | 28.5485 | 147 | 0.3689±0.0563 | 6.0056±0.3383 | 0.2541±0.0084 |  | 0.3104±0.0086 | 5.4860±0.1223 | 0.3290±0.0078 |
| 61 | **Allose** | Sugars | 28.8253 | 76 | 0.1365±0.0524 | 0.1658±0.0070 | 1.0333±0.0561 |  | 0.0056±0.0002 | 0.1189±0.0024 | 1.0085±0.0538 |
| 62 | **D-Fructose** | Sugars | 29.0892 | 307 | 0.8363±0.1005 | 20.2658±0.8518 | 0.8521±0.0756 |  | 0.6250±0.0174 | 15.2038±0.3318 | 0.7409±0.0456 |
| 63 | D-Talose | Sugars | 29.3917 | 321 | 0.4510±0.0669 | -- | -- |  | 0.1882±0.0052 | -- | -- |
| 64 | D-Galactose | Sugars | 29.6428 | 244 | -- | 3.8923±0.8067 | -- |  | -- | 0.5762±0.0121 | -- |
| 65 | **D-Glucose** | Sugars | 29.7700 | 103 | -- | 39.3392±1.7414 | 3.0726±0.1717 |  | -- | 31.9585±0.3590 | 2.9400±0.1256 |
| 66 | Sorbitol | Alcohols | 29.8037 | 321 | 0.2619±0.0480 | -- | -- |  | 0.1354±0.0038 | -- | -- |
| 67 | **β-D-Glucopyranose** | Sugars | 30.1063 | 129 | -- | 0.2730±0.0179 | 0.0445±0.0022 |  | -- | 0.1830±0.0055 | 0.1060±0.0041 |
| 68 | **Ascorbic acid** | Organic acids | 30.2994 | 117 | -- | 0.0172±0.0010 | 0.0028±0.0003 |  | -- | 0.0234±0.0005 | 0.0031±0.0001 |
| 69 | **Gallic acid** | Organic acids | 30.5375 | 191 | -- | 3.5545±0.2840 | 0.4058±0.0103 |  | -- | 2.2554±0.0612 | 0.5091±0.0120 |
| 70 | Glucopyranoside | Sugars | 30.9816 | 191 | 0.0043±0.0002 | -- | -- |  | 0.0042±0.0001 | -- | -- |
| 71 | Hydroferulic acid | Organic acids | 31.4772 | 147 | 0.0009±0.0001 | 0.1017±0.0048 | -- |  | 0.0008±0.0000 | 0.0778±0.0020 | -- |
| 72 | **Palmitic acid** | Organic acids | 32.2947 | 117 | 0.0508±0.0082 | 1.0396±0.0419 | 0.1315±0.0060 |  | 0.0207±0.0006 | 0.8719±0.0091 | 0.1114±0.0049 |
| 73 | 10-Heptadecenoic acid | Organic acids | 32.6101 | 55 | -- | 0.1520±0.0062 | -- |  | -- | 0.1298±0.0023 | -- |
| 74 | Mannitol | Alcohols | 32.8483 | 43 | -- | -- | 0.0153±0.0007 |  | -- | -- | 0.0134±0.0003 |
| 75 | **Myo-Inositol** | Alcohols | 32.9963 | 147 | 0.3502±0.0352 | 5.6559±0.3181 | 0.2672±0.0090 |  | 0.2598±0.0072 | 3.5818±0.0712 | 0.3246±0.0112 |
| 76 | **Galactitol** | Alcohols | 33.7300 | 73 | 0.0064±0.0006 | 1.0409±0.2365 | 0.0028±0.0003 |  | 0.0061±0.0002 | 0.4620±0.0235 | 0.0053±0.0006 |
| 77 | **N-Acetyl-D-glucosamine** | Amines | 33.9875 | 103 | 0.0006±0.0001 | 0.4846±0.1016 | 0.0045±0.0006 |  | 0.0010±0.0000 | 0.2358±0.0117 | 0.0097±0.0013 |
| 78 | **Linoleic acid** | Organic acids | 35.2426 | 55 | 0.0069±0.0010 | 0.1000±0.0064 | 0.0182±0.0008 |  | 0.0025±0.0001 | 0.0549±0.0011 | 0.0142±0.0006 |
| 79 | Petroselinic acid | Organic acids | 35.3521 | 129 | -- | 0.1712±0.0080 | 0.1304±0.0066 |  | -- | 0.1303±0.0024 | 0.1016±0.0044 |
| 80 | Oleic acid | Organic acids | 35.4873 | 129 | -- | 0.0246±0.0010 | 0.0121±0.0006 |  | -- | 0.0207±0.0004 | 0.0090±0.0005 |
| 81 | **Floridoside** | Sugars | 34.5604 | 131 | 0.0002±0.0000 | 1.3870±0.0680 | 0.0275±0.0016 |  | 0.0003±0.0000 | 0.8179±0.0270 | 0.0939±0.0036 |
| 82 | **Stearic acid** | Organic acids | 35.8348 | 117 | 0.0095±0.0016 | 0.1412±0.0057 | 0.0151±0.0005 |  | 0.0043±0.0001 | 0.0891±0.0009 | 0.0139±0.0005 |
| 83 | **Glyceryl-glycoside** | Sugars | 36.9677 | 103 | 0.0038±0.0002 | 0.1770±0.0153 | 0.0059±0.0005 |  | 0.0031±0.0001 | 0.1002±0.0052 | 0.0181±0.0007 |
| 84 | 9-Octadecenamide | Amines | 37.8752 | 59 | 0.0083±0.0007 | 0.1416±0.0085 | -- |  | 0.0052±0.0001 | 0.0756±0.0032 | -- |
| 85 | **D-Glucuronic acid** | Organic acids | 37.9975 | 73 | 0.0741±0.0060 | 1.2212±0.0898 | 0.0430±0.0008 |  | 0.0869±0.0024 | 0.7277±0.0316 | 0.0508±0.0011 |
| 86 | Adenosine | Others | 41.9497 | 236 | -- | -- | 0.0145±0.0007 |  | -- | -- | 0.0093±0.0005 |
| 87 | **Sucrose** | Sugars | 42.0269 | 361 | 2.4925±0.3663 | 18.2566±0.9238 | 0.6510±0.0096 |  | 1.5739±0.0437 | 11.9082±0.0884 | 0.6714±0.0067 |
| 88 | Lactulose | Sugars | 42.2972 | 103 | 0.1060±0.0090 | -- | 0.0147±0.0003 |  | 0.1015±0.0028 | -- | 0.0171±0.0002 |
| 89 | **3-α-Mannobiose** | Sugars | 42.5225 | 191 | 0.0084±0.0005 | 0.1062±0.0063 | 0.0069±0.0004 |  | 0.0087±0.0002 | 0.0765±0.0019 | 0.0046±0.0002 |
| 90 | **Cellobiose** | Sugars | 42.8636 | 191 | 0.0014±0.0002 | 0.0782±0.0066 | 0.0128±0.0012 |  | 0.0009±0.0000 | 0.0314±0.0007 | 0.0055±0.0004 |
| 91 | **D-Maltose** | Sugars | 43.5588 | 191 | 0.0227±0.0017 | 0.0841±0.0061 | 0.0190±0.0011 |  | 0.0167±0.0005 | 0.0409±0.0007 | 0.0155±0.0003 |
| 92 | **Turanose** | Sugars | 43.7197 | 73 | 0.0305±0.0024 | 0.3065±0.0420 | 0.0178±0.0005 |  | 0.0315±0.0009 | 0.2099±0.0284 | 0.0270±0.0012 |
| 93 | 2-α-Mannobiose | Sugars | 44.0995 | 103 | 0.0028±0.0003 | 0.2014±0.0119 | -- |  | 0.0025±0.0001 | 0.1323±0.0025 | -- |
| 94 | Gentiobiose | Sugars | 44.7109 | 73 | 0.0067±0.0004 | 0.0213±0.0006 | -- |  | 0.0058±0.0002 | 0.0222±0.0003 | -- |
| 95 | Palatinose | Sugars | 44.8139 | 73 | 0.0022±0.0005 | -- | 0.0160±0.0018 |  | 0.0055±0.0002 | -- | 0.0235±0.0023 |
| 96 | **Catechin** | Organic acids | 45.5091 | 73 | 0.0075±0.0015 | 0.5052±0.0941 | 0.0767±0.0056 |  | 0.0036±0.0001 | 0.7268±0.0385 | 0.0426±0.0034 |
| 97 | Galactinol | Alcohols | 46.8350 | 147 | 0.0084±0.0022 | 0.0242±0.0017 | -- |  | 0.0032±0.0001 | 0.0422±0.0020 | -- |
| 98 | **Tocopherol** | Alcohols | 47.0539 | 43 | 0.0008±0.0001 | 0.0440±0.0029 | 0.0052±0.0002 |  | 0.0006±0.0000 | 0.0426±0.0011 | 0.0062±0.0001 |
| 99 | **Melibiose** | Sugars | 48.7596 | 129 | 0.0017±0.0001 | 0.0858±0.0049 | 0.0065±0.0002 |  | 0.0015±0.0000 | 0.0606±0.0010 | 0.0083±0.0003 |
| 100 | β-Sitosterol | Alcohols | 51.6046 | 43 | -- | 0.7225±0.0473 | 0.0174±0.0003 |  | -- | 0.3551±0.0051 | 0.0171±0.0003 |

--, not determined. All data are mean±SE (in July, n = 4 of P-rich sites, n = 5 of P-deficient sites; in August and September, n = 8 of P-rich sites, of P-deficient sites, n = 12).
