# Supplementary material for: Roles of metabolic regulation in developing Quercus variabilis acorns at contrasting geologically-derived phosphorus sites in subtropical China
Source: BMC Plant Biol. 2020 Aug 25;20:389. doi: 10.1186/s12870-020-02605-y (PMC7449008; doi:10.1186/s12870-020-02605-y)
Supplement: Supplementary file 5 — Additional file 5: Table S2. Results of pathway analysis involving all identified metabolites in acorns during three developmental stages. All pathways shown in the table are potential target metabolic pathways with pathway impacts of above 0.1. [file 12870_2020_2605_MOESM5_ESM.doc]

**Table S2 Results of pathway analysis involving all identified metabolites in acorns during three developmental stages**

| **Stage** | **Pathway** | **Total** | **Expected** | **Hits** | **Holm adjust** | **FDR** | **Impact** |
| --- | --- | --- | --- | --- | --- | --- | --- |
| **Jul.** | Galactose metabolism | 26 | 1.25 | 7 | 0.01 | 0.01 | 0.14 |
|  | Starch and sucrose metabolism | 30 | 1.44 | 3 | 1.00 | 1.00 | 0.11 |
|  | Pentose and glucuronate interconversions | 12 | 0.58 | 2 | 1.00 | 1.00 | 0.20 |
|  | Alanine, aspartate and glutamate metabolism | 22 | 1.06 | 3 | 1.00 | 1.00 | 0.33 |
|  | Glycine, serine and threonine metabolism | 30 | 1.44 | 3 | 1.00 | 1.00 | 0.35 |
|  | Arginine and proline metabolism | 38 | 1.83 | 3 | 1.00 | 1.00 | 0.16 |
|  | β-Alanine metabolism | 12 | 0.58 | 1 | 1.00 | 1.00 | 0.54 |
|  | Pantothenate and CoA biosynthesis | 14 | 0.67 | 2 | 1.00 | 1.00 | 0.20 |
|  | Glyoxylate and dicarboxylate metabolism | 17 | 0.82 | 2 | 1.00 | 1.00 | 0.11 |
|  | Inositol phosphate metabolism | 24 | 1.16 | 2 | 1.00 | 1.00 | 0.25 |
| **Aug.** | Galactose metabolism | 26 | 1.29 | 7 | 0.01 | 0.01 | 0.16 |
|  | Starch and sucrose metabolism | 30 | 1.49 | 4 | 1.00 | 1.00 | 0.18 |
|  | Pentose and glucuronate interconversions | 12 | 0.6 | 2 | 1.00 | 1.00 | 0.20 |
|  | Alanine, aspartate and glutamate metabolism | 22 | 1.09 | 3 | 1.00 | 1.00 | 0.53 |
|  | Arginine and proline metabolism | 38 | 1.89 | 3 | 1.00 | 1.00 | 0.14 |
|  | Glycine, serine and threonine metabolism | 30 | 1.49 | 2 | 1.00 | 1.00 | 0.22 |
|  | Glyoxylate and dicarboxylate metabolism | 17 | 0.84 | 2 | 1.00 | 1.00 | 0.11 |
|  | Inositol phosphate metabolism | 24 | 1.19 | 2 | 1.00 | 1.00 | 0.25 |
|  | Ascorbate and aldarate metabolism | 15 | 0.75 | 3 | 1.00 | 1.00 | 0.18 |
| **Sept.** | Galactose metabolism | 26 | 1.25 | 6 | 0.09 | 0.09 | 0.12 |
|  | Starch and sucrose metabolism | 30 | 1.44 | 4 | 1.00 | 0.76 | 0.18 |
|  | Pentose and glucuronate interconversions | 12 | 0.58 | 2 | 1.00 | 1.00 | 0.20 |
|  | Alanine, aspartate and glutamate metabolism | 22 | 1.06 | 4 | 1.00 | 0.71 | 0.33 |
|  | Arginine and proline metabolism | 38 | 1.83 | 3 | 1.00 | 1.00 | 0.16 |
|  | Glycine, serine and threonine metabolism | 30 | 1.44 | 3 | 1.00 | 1.00 | 0.35 |
|  | Ascorbate and aldarate metabolism | 15 | 0.72 | 3 | 1.00 | 0.71 | 0.18 |
|  | Glyoxylate and dicarboxylate metabolism | 17 | 0.82 | 2 | 1.00 | 1.00 | 0.11 |
|  | Citrate cycle (TCA cycle) | 20 | 0.96 | 2 | 1.00 | 1.00 | 0.14 |
|  | Inositol phosphate metabolism | 24 | 1.16 | 2 | 1.00 | 1.00 | 0.25 |

All pathways shown in the table are potential target metabolic pathways with pathway impacts above 0.1.
