# Supplementary material for: Roles of metabolic regulation in developing Quercus variabilis acorns at contrasting geologically-derived phosphorus sites in subtropical China
Source: BMC Plant Biol. 2020 Aug 25;20:389. doi: 10.1186/s12870-020-02605-y (PMC7449008; doi:10.1186/s12870-020-02605-y)
Supplement: Supplementary file 6 — Additional file 6: Table S3. Concentrations of acorn elements in September at P-rich and P-deficient sites (mg/g). All data are mean ± SE (n = 8 at P-rich sites; n = 12, at P-deficient sites). Discriminating elements, with bold text for p value and VIP (variable importance projection plot) value, were selected based on the p < 0.05 from the t test (Significance, p < 0.05) and VIP > 1 from OPLS-DA models. [file 12870_2020_2605_MOESM6_ESM.doc]

**Table S3** Concentrations of acorn elements in September at P-rich and P-deficient sites (mg g-1)

| **Elements** | **P-rich sites** | **P-deficient sites** | ***p* value** | **VIP** |
| --- | --- | --- | --- | --- |
| C | 417.22±0.25 | 416.68±0.20 | 0.61 | < 1.00 |
| H | 63.37±0.05 | 63.15±0.16 | 0.76 | < 1.00 |
| O | 483.85±0.69 | 478.72±0.40 | 0.04 | < 1.00 |
| N | 4.55±0.05 | 3.50±0.03 | **0.00** | **2.02** |
| P | 0.70±0.01 | 0.62±0.00 | **0.01** | **1.28** |
| K | 4.57±0.12 | 4.53±0.03 | 0.91 | < 1.00 |
| Ca | 0.41±0.01 | 0.37±0.02 | 0.63 | < 1.00 |
| Mg | 0.48±0.01 | 0.43±0.01 | 0.22 | < 1.00 |
| S | 0.37±0.01 | 0.28±0.01 | **0.00** | **1.48** |
| Fe | 0.02±0.00 | 0.06±0.01 | 0.46 | 1.22 |
| Al | 0.01±0.00 | 0.02±0.00 | 0.38 | < 1.00 |
| Mn | 0.07±0.00 | 0.19±0.01 | **0.00** | **1.73** |
| Na | 0.03±0.00 | 0.10±0.03 | 0.55 | 1.61 |
| Zn | 0.00±0.00 | 0.01±0.00 | 0.48 | < 1.00 |
| Cu | 0.01±0.00 | 0.00±0.00 | **0.02** | **1.51** |

All data are mean±SE (n = 8 at P-rich sites; n = 12, at P-deficient sites). Discriminating elements, with bold text for *p* value and VIP (variable importance projection plot) value, are selected based on the *p* < 0.05 from the *t* test (Significance, *p* < 0.05) and VIP > 1 from OPLS-DA models.
