# Supplementary material for: Roles of metabolic regulation in developing Quercus variabilis acorns at contrasting geologically-derived phosphorus sites in subtropical China
Source: BMC Plant Biol. 2020 Aug 25;20:389. doi: 10.1186/s12870-020-02605-y (PMC7449008; doi:10.1186/s12870-020-02605-y)
Supplement: Supplementary file 7 — Additional file 7: Table S4. Correlations (r, Pearson’s correlation coefficient) between acorn discriminating elements between P-rich and P-deficient sites in September. Significance, p value < 0.05: *, p < 0.05; **, p < 0.01. [file 12870_2020_2605_MOESM7_ESM.doc]

**Table S4** Correlations (*r*, Pearson's correlation coefficient) between acorn discriminating elements between P-rich and P-deficient sites in September

| r | N | P | S | Mn | Cu |
| --- | --- | --- | --- | --- | --- |
| N | 1 | 0.62^**^ | 0.63^**^ | -0.56^*^ | 0.73^**^ |
| P | 0.62^**^ | 1 | 0.77^**^ | -0.08 | 0.41 |
| S | 0.63^**^ | 0.77^**^ | 1 | -0.09 | 0.52^*^ |
| Mn | -0.56^*^ | -0.08 | -0.09 | 1 | -0.45^*^ |
| Cu | 0.73^**^ | 0.41 | 0.52^*^ | -0.45^*^ | 1 |

Significance, *p* value < 0.05: **^*^**, *p* < 0.05; **^**^**, *p* < 0.01.
