# Supplementary material for: Roles of metabolic regulation in developing Quercus variabilis acorns at contrasting geologically-derived phosphorus sites in subtropical China
Source: BMC Plant Biol. 2020 Aug 25;20:389. doi: 10.1186/s12870-020-02605-y (PMC7449008; doi:10.1186/s12870-020-02605-y)
Supplement: Supplementary file 8 — Additional file 8: Table S5. Correlations (r, Pearson’s correlation coefficient) between differential metabolites and discriminating elements in acorns between P-rich and P-deficient sites in September. Significance, p value < 0.05: *, p < 0.05; **, p < 0.01. [file 12870_2020_2605_MOESM8_ESM.doc]

**Table S5** Correlations (*r*, Pearson's correlation coefficient) between differential metabolites and discriminating elements in acorns between P-rich and P-deficient sites in September

| *r* | N | P | S | Mn | Cu |
| --- | --- | --- | --- | --- | --- |
| Glycine | 0.49^*^ | 0.65^**^ | 0.48^*^ | -0.36 | 0.15 |
| D-Serine | 0.38 | 0.22 | 0.25 | -0.37 | 0.06 |
| γ-Aminobutyric acid | 0.42 | 0.26 | 0.17 | -0.49^*^ | 0.07 |
| Erythrose | -0.32 | -0.22 | -0.15 | -0.06 | -0.15 |
| Lactulose | -0.41 | -0.25 | -0.35 | 0.26 | -0.46^*^ |
| Cellobiose | 0.51^*^ | 0.18 | -0.01 | -0.55^*^ | 0.32 |
| L-Sorbofuranose | -0.54^*^ | -0.15 | -0.26 | 0.36 | -0.16 |
| β-D-Glucopyranose | -0.51^*^ | -0.34 | -0.44 | 0.61^**^ | -0.50^*^ |
| Floridoside | -0.72^**^ | -0.58^**^ | -0.56^*^ | 0.64^**^ | -0.47^*^ |
| Orthophosphoric acid | 0.64^**^ | 0.24 | 0.38 | -0.61^**^ | 0.24 |
| Ribonic acid | -0.50^*^ | -0.15 | -0.27 | 0.55^*^ | -0.22 |
| Glyceryl-glycoside | -0.68^**^ | -0.57^**^ | -0.59^**^ | 0.55^*^ | -0.56^*^ |
| Ethylene glycol | -0.45^*^ | -0.19 | -0.37 | 0.46^*^ | -0.05 |
| 1,2,3-Butanetriol | -0.51^*^ | -0.46^*^ | -0.46^*^ | 0.42 | -0.25 |
| Ethanolamine | 0.42 | 0.41 | 0.33 | -0.41 | -0.08 |

Significance, *p* value < 0.05: **^*^**, *p* < 0.05; **^**^**, *p* < 0.01.
